# Radiation-enhanced therapeutic targeting of galectin-1 enriched malignant stroma in triple negative breast cancer

**DOI:** 10.18632/oncotarget.9490

**Published:** 2016-05-19

**Authors:** Meenakshi Upreti, Amar Jyoti, Sara E. Johnson, Elden P. Swindell, Dana Napier, Pallavi Sethi, Ryan Chan, Jonathan M. Feddock, Heidi L. Weiss, Thomas V. O'Halloran, B. Mark Evers

**Affiliations:** ^1^ Department of Pharmaceutical Sciences, College of Pharmacy, University of Kentucky, Lexington, KY, USA; ^2^ Markey Cancer Center, University of Kentucky, Lexington, KY, USA; ^3^ Department of Pathology, University of Kentucky, Lexington, KY, USA; ^4^ Department of Chemistry, Chemistry of Life Processes Institute, Northwestern University, Evanston, IL, USA; ^5^ Department of Radiation Medicine, University of Kentucky Chandler Hospital, Lexington, KY, USA; ^6^ Department of Surgery, University of Kentucky, Lexington, KY, USA

**Keywords:** galectin-1, triple negative breast cancer, stromal-targeting, TNBC tumor model, tumor tissue analog (TTA)

## Abstract

Currently there are no FDA approved targeted therapies for Triple Negative Breast Cancer (TNBC). Ongoing clinical trials for TNBC have focused primarily on targeting the epithelial cancer cells. However, targeted delivery of cytotoxic payloads to the non-transformed tumor associated-endothelium can prove to be an alternate approach that is currently unexplored. The present study is supported by recent findings on elevated expression of stromal galectin-1 in clinical samples of TNBC and our ongoing findings on stromal targeting of radiation induced galectin-1 by the anginex-conjugated arsenic-cisplatin loaded liposomes using a novel murine tumor model. We demonstrate inhibition of tumor growth and metastasis in response to the multimodal nanotherapeutic strategy using a TNBC model with orthotopic tumors originating from 3D tumor tissue analogs (TTA) comprised of tumor cells, endothelial cells and fibroblasts. The ‘rigorous’ combined treatment regimen of radiation and targeted liposomes is also shown to be well tolerated. More importantly, the results presented provide a means to exploit clinically relevant radiation dose for concurrent receptor mediated enhanced delivery of chemotherapy while limiting overall toxicity. The proposed study is significant as it falls in line with developing combinatorial therapeutic approaches for stroma-directed tumor targeting using tumor models that have an appropriate representation of the TNBC microenvironment.

## INTRODUCTION

Breast cancer (BC) is the second leading cause of death in women. Of those diagnosed with breast cancer, 15–20% are classified as triple negative breast cancer (TNBC) [1, 2] prevalent in women with African American ethnicity and younger age [3–5]. TNBC is a heterogeneous subtype, that is histopathologically diagnosed based on the characteristics of “triple negativity” defined by the lack of expression or overexpression of the “three receptors': estrogen receptor (ER), progesterone receptor (PR) and the epidermal growth factor receptor type 2 (HER2) [6]. More aggressive than other forms of breast cancer, TNBC presents an early, truculent visceral metastases, a short life expectancy with poor prognosis and few effective treatment options [3, 7–9]. The standard of care for TNBC patients' remains to be traditional chemotherapy, surgery and radiation. Unlike the receptor driven breast cancers, the oncologists do not have any FDA approved targeted therapies from the existing drug arsenal for advanced stage of TNBC that presents an average progression free survival of 12 months, which is far less than the other subtypes of BC. Identifying specific targets for more effective and promising therapies for treatment of TNBC has therefore become a major clinical challenge. An improved understanding of the biology of TNBC has led to the discovery of therapeutic targets for TNBC, such as the Notch signaling pathway (RO-4929097) [10], Wnt/b-catenin pathway (Salinomycin) [11], DNA repair pathways (PARP1 inhibitors: Iniparib, Olaparib) [12, 13], Overexpressed epidermal growth factor (EGFR inhibitors: Gefltinib, cetuximab) [14, 15], P13/Akt/mammalian target of rapamycin (mTOR) activation (mTOR inhibitors (Everolimus) [16] and TGF-β signaling pathway (TGFBR1 inhibitor: LY2157299) [17]. However, the molecular heterogeneity of TNBC [18, 19] may explain for the marginal benefits in clinical trials with these targeted therapies that are majorly focused on targeting single pathways in the epithelial cancer cells, a critical contributor to the TNBC heterogeneity [20]. The limited success of the molecularly targeted therapies in TNBC thus, highlights the ongoing importance for cytotoxic therapy and the need for ‘think-out of the box’ approaches to develop novel treatment strategies augmenting the current treatment options for TNBC.

Therapeutic options to target the tumor stroma are limited with the most exploited exception being bevacizumab (Avastin, Roche), a monoclonal antibody that targets vascular endothelial growth factor (VEGF) and inhibits tumor angiogenesis [21]. However, its use in the treatment of TNBC has not been encouraging and therefore was restricted by the FDA in late 2011 for subsequent use in the treatment of metastatic breast cancer [22]. A carbohydrate binding protein, galectin-1, which plays a pivotal role in tumor growth and cancer progression including invasion, angiogenesis and metastasis is a propitious alternative for stromal targeting of the non-transformed tumor associated-endothelium. Studies, including our own, have demonstrated the potential of galectin-1 as a therapeutic target by identifying the overexpression of galectin-1 in various human cancers and enrichment in tumor stroma [23, 24]. In addition, we have reported a further induction of galectin-1 expression in tumor stroma as a response to radiation therapy, suggesting that targeting galectin-1 may synergize with radiation therapy [23–25]. A number of novel compounds and approaches to block galectin-1 or its activity are currently being evaluated [23]. The synthetic antiangiogenic peptide, anginex, has been shown to bind specifically to the galectin-1 receptor and delay the tumor growth [26–28]. Anginex is an antiangiogenic 33 amino acid beta sheet peptide [29–31] that specifically binds and inhibits the function of galectin-1 receptor [27, 32–34]. While unable to completely block tumor growth, anginex can serve as an excellent ligand for targeted delivery of liposomal cytotoxic payloads to the tumor endothelium. However, developing nanotherapeutic regimens that target the tumor stroma implicitly demand reliable tumor models that replicate the stromal characteristics of the human cancer. Using a murine *in vitro/in vivo* TNBC tumor model system with galectin-1 enriched stroma we have reported that conjugation of anginex to liposomes enables preferential targeting of the unique mix of dual chemotherapy released at the irradiated tumor endothelium in a controlled manner over time, beyond that expected from enhanced tumor permeability and retention [25]. This novel therapeutic approach is designed to destabilize the tumor microenvironment which sensitizes and primes the tumor cells for cell death, maximizing the therapeutic gain of the antitumor strategy in selective and effective inhibition of tumor growth and metastasis in contrast to conventional systemic approaches. In this study we establish the overexpression of galectin-1 in the TNBC stroma using the murine tumor model system with an appropriate representation of the tumor microenvironment developed in our laboratory we have investigated the therapeutic efficacy as well as the biocompatibility of the radiation enhanced galectin-1 targeting with anginex conjugated arsenic trioxide (ATO) and cisplatin loaded liposomes to the TNBC microenvironment.

## RESULTS

### Similarity in the expression profile of galectin-1 to VEGF-A in ductal carcinoma of the breast indicate it's potential as a candidate for stroma-directed molecular targeting

**Figure 1:**
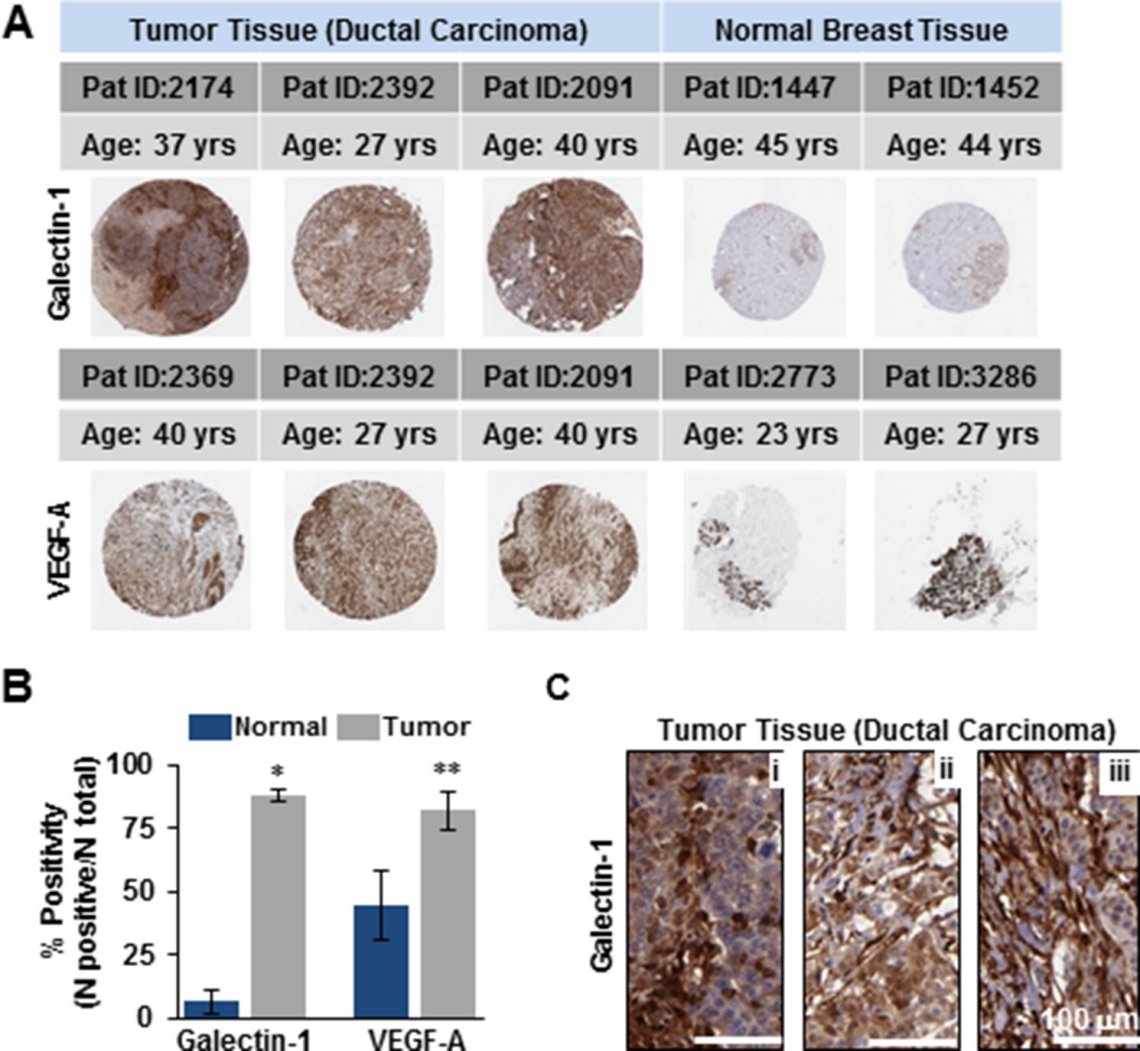
Elevated levels of galectin-1 in human tissues of ductal carcinoma compared to the normal breast tissue resemble the expression profile of VEGF-A (**A**) Spot images of human breast cancer (ductal carcinoma) and normal breast tissues 1 mm in diameter of women 27–40 years in age stained with galectin-1 antibody (Sigma) [Upper panel] and VEGF antibody (Santa Cruz Biotech.) [Lower panel]. (**B**) Quantitation of image scans (Aperioscope) demonstrating higher % of positive staining for both galectin-1 and VEGF-A in ductal carcinoma versus normal breast tissue with *p < 0.0001 and **p < 0.05 respectively (mean ± SE; *n* = 3 samples/group). (**C**) 20X magnification of ductal carcinoma sections in (A) immunoprobed for galectin-1 demonstrating its stromal enrichment (Brown). Tumor cells stain blue (DAPI). Data were provided by the human protein atlas database. Scale bar = 100 μm.

**Figure 2:**
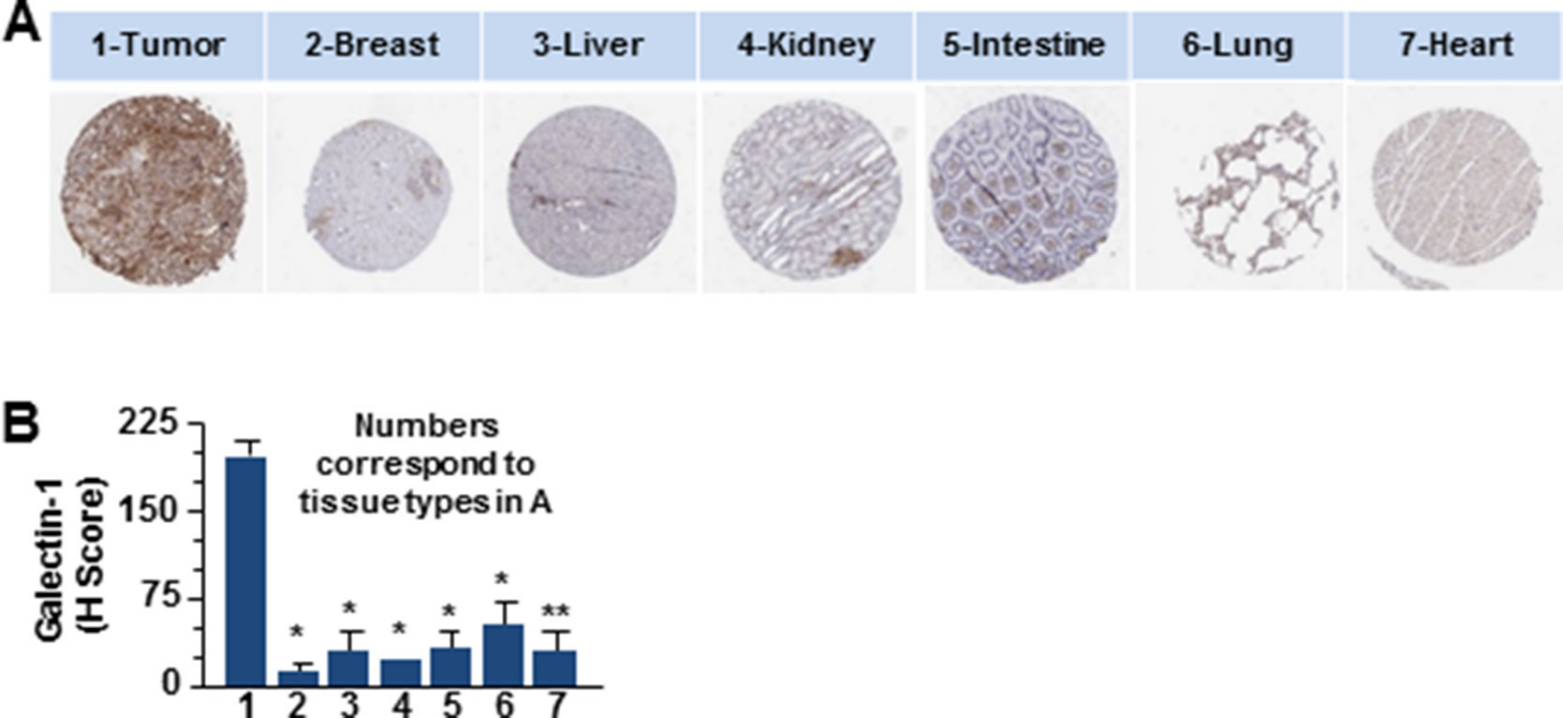
The differential expression profile of galectin-1 in various human tissues compared to ductal carcinoma (**A**) Representative spot images of six types of tissues 1 mm in diameter as indicated were probed (Brown) for the expression of galectin-1 (Sigma) were compared to tumor tissue of ductal carcinoma stained for galectin-1. Data were provided by the human protein atlas database (http://www.proteinatlas.org/). (**B**) Galectin-1 staining was quantitated by APERIO^®^ ImageScope and scored using the H-score approach that combines the pixel intensity with the percentage of tissue as described in the methods and graphically represented. (*N* = 3, ^*^p < 0.005 or ^**^p < 0.05, for tissues from the tumor compared to that from other organs).

The primary objective of targeted drug nanoparticle delivery is to isolate the disease *in vivo* against the background of the normal functioning tissue without disrupting or altering usual physiologic processes. The task of developing such a receptor-targeted nanoparticle delivery is particularly challenging as it depends upon identifying a biomarker or disease characteristic that can be ‘targeted’ to achieve the goal. There is compelling evidence for galectin-1 as an important protein in cancer biology that is enriched in the tumor- associated neovascular endothelium [24, 27, 32, 33]. We also explored the data from The Human Protein Atlas for the expression of galectin-1 in ductal carcinomas of women between 27–40 years of age (Figure 1A). Statistical evaluation of the immunohistochemistry revealed significantly increased expression of galectin-1 in tumor tissues as compared to the normal breast tissues (Figure 1B). Enlarged images of the ductal carcinoma tissue sections (20×) demonstrated the stromal enrichment of galectin-1 (Figure 1B). Furthermore, this difference in the expression of galectin-1 between normal versus the ductal carcinoma tissues of patients was found to be comparable to the expression of vascular endothelial growth factor (VEGF-A) in the same or similar tissues. VEGF-A, a multifunctional cytokine secreted by human tumors, is implicated with poor prognosis in breast cancer [35, 36]. One of the major challenges in developing a molecular targeted therapy is in identifying a biomarker significantly overexpressed in the tumor tissue, not just in comparison to the corresponding normal tissue but in tissue of other normal organs. The data from the Human Protein Atlas in Figure 2 demonstrates that while galectin-1 is expressed in organs other than the breast, the quantitation of representative images indicated the galectin-1 expression to be notably higher in the tumor tissues in comparison to the other normal tissues (Figure 2).

### Elevated expression of stromal galectin-1 in clinical samples of TNBC

**Figure 3:**
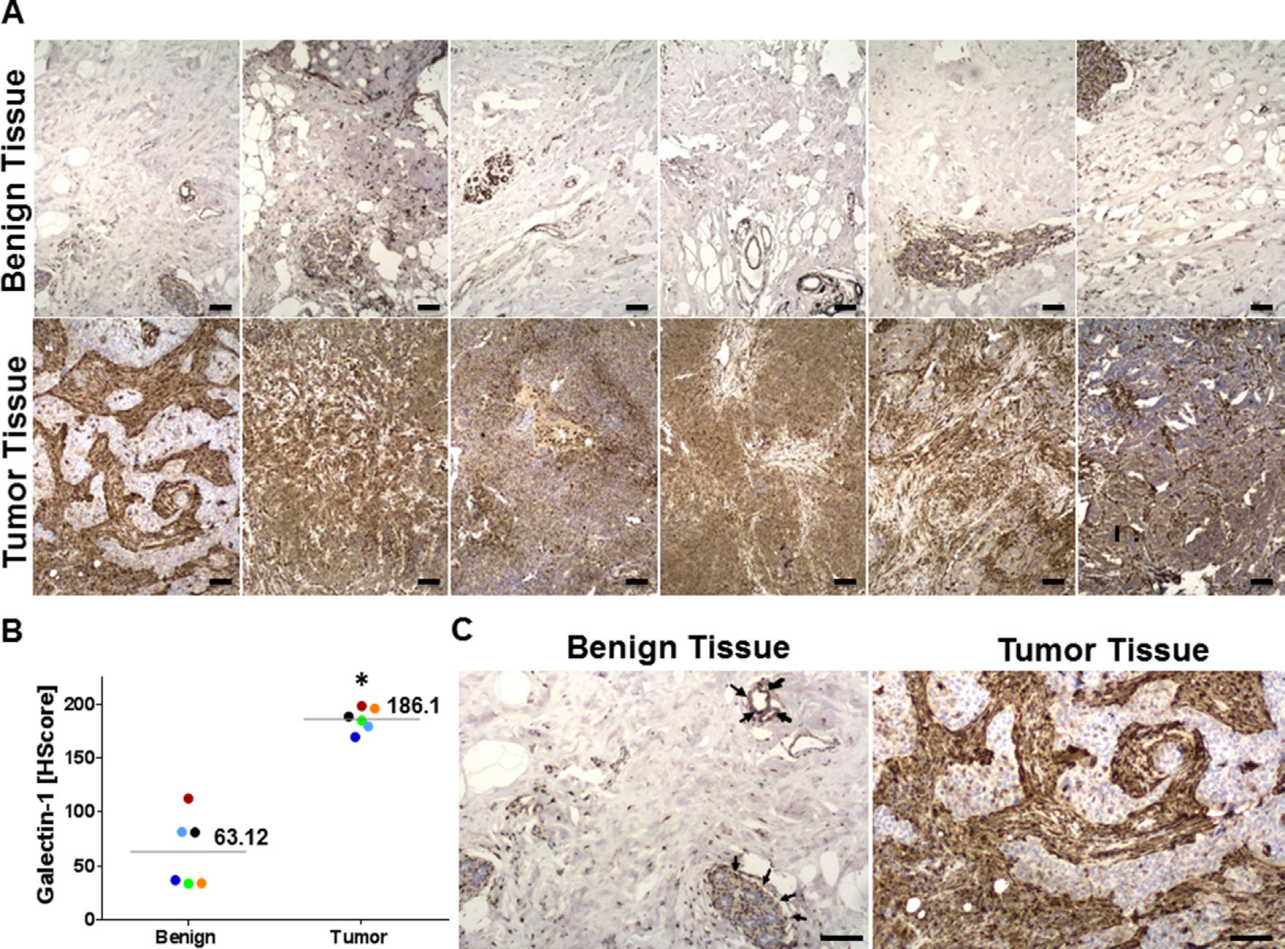
Galectin-1 overexpression in clinical samples of triple negative breast cancer (TNBC) patients (**A**) Immunohistochemistry of six cases of TNBC matched with the corresponding normal breast tissues for galectin-1. The tissue sections were stained with anti-galectin-1 antibody (sigma) and counterstained with Hematoxylin and Eosin (Supplementary Figure S1) at 100X magnification. Scale bar = 25 μm (**B**) Galectin-1 staining in each histological section of benign and tumor tissues of the six TNBC patients was scored using the H-score approach as described in the methods and graphically represented. The percentage of tissue stained was quantitated by APERIO^®^ ImageScope (*N* = 6, *p = 0.0002 for tumor tissue compared to the benign tissue). (**C**) Representative images of benign and primary tumor tissue probed for galectin-1. While the tumor cells stained weakly (blue/light brown), the tumor associated stroma stained strongly for galectin-1 (brown) in the tumor tissue. The myoepithelial cells/clusters and ductal/lobular units in the benign breast tissue also stained strongly for galectin-1 (black arrows) but the staining was restricted to distinct foci. Scale bar = 50 μm.

Galectin-1 staining in normal breast tissue was identified using immunohistochemical analysis, and there appeared to be a low level of membrane staining primarily involving the normal ductal and lobular units, with no significant staining of the myoepithelial or adipose tissue (black arrows) (Figure 3A and 3C). The median H score for Galectin-1 for benign tissue was 63.12. In contrast, tumor staining was more uniform with a more intense membrane staining pattern in both the ductal and lobular units. The pronounced staining in the tumors was in the intervening myoepithelial and stromal tissue, with a median H Score of 186.1 (Figure 3A–3B). This suggests a significant difference in the level of galectin-1 expression between benign and tumor tissue that could be manipulated as a target for tumor specific drug delivery. Histopathology of the tumor and benign tissue sections from the TNBC patient samples was evidenced by the hematoxylin and eosin (H&E) staining (Supplementary Figure S1).

### Radiation exposure augments the expression of galectin-1 in the murine TNBC model developed from orthotopic implant of 3D ‘Tumor tissue analog’ (TTA) in nude mice that better represents the malignant stroma

**Figure 4:**
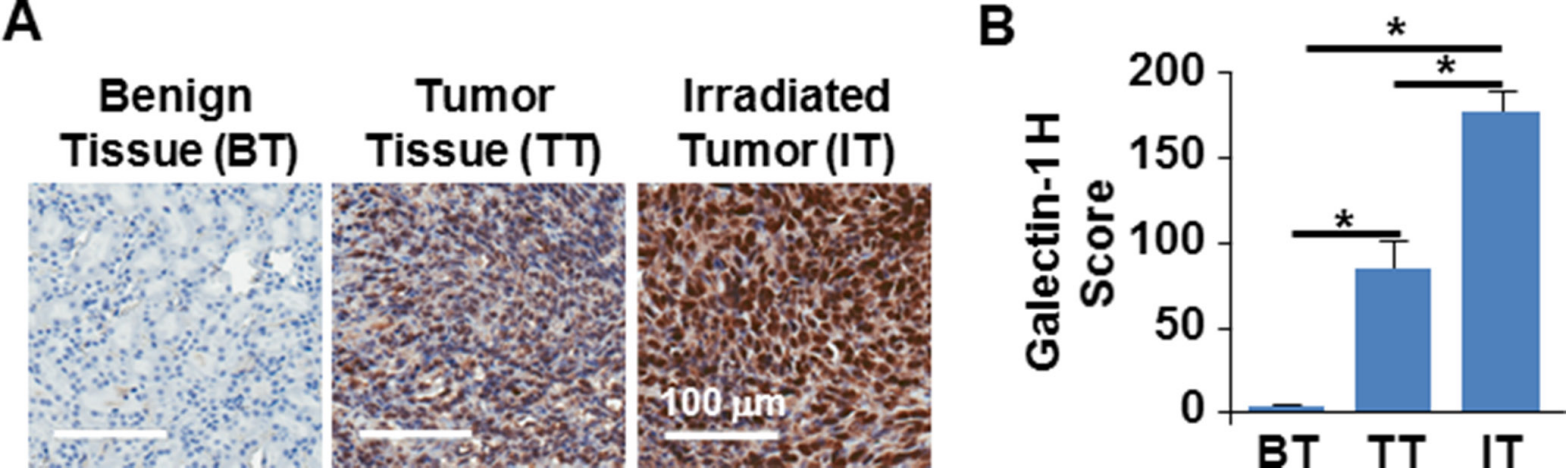
Radiation augmented galectin-1 expression in orthotopic tumors originating from TTA (**A**) Representative images of immunohistochemistry for galectin-1 in benign breast tissue (BT), tumor tissue (TT) and irradiated tumor tissue (IT) originating from orthotopic implants of TTA in mice 72 hours post-radiation exposure of 2 Gy. (**B**) Galectin-1 staining was quantitated by APERIO^®^ ImageScope and scored using the H-score approach as described in the methods and graphically represented. *N* = 3/group, *p < 0.0003 for TT vs IT, BT vs. IT and BT vs TT.

We have demonstrated the usefulness of the 3D TNBC model in *in vivo* setting by implantation of the TTA in the mammary fat pad of nude mice [24, 25]. This preclinical tumor model incorporates aspects of the tumor microenvironment and the neovascular architecture, critical for evaluating nanoparticles and stroma-directed molecular targeting. Our studies have also elucidated the radiation-induced galectin-1 surge to be more pronounced in the murine TNBC model developed from orthotopic implants of TTA comprised of tumor cells, endothelial cells and fibroblasts than the orthotopic implants of tumor cell only–spheroids [25]. Using the same murine TNBC model in this study we compare the galectin-1 expression between benign breast, tumor and irradiated tumor tissue originating from TTA implants in the mammary fat pad of nude mice (Figure 4A and Supplementary Figure S2). The H-scores for galectin-1 expression were found to be significantly higher in the irradiated tumor tissue in comparison to the non-irradiated or benign breast tissue (Figure 4B).

### Sensitivity to ATO and cisplatin cytotoxicity in cell types is associated with p53 phosphorylation

**Figure 5:**
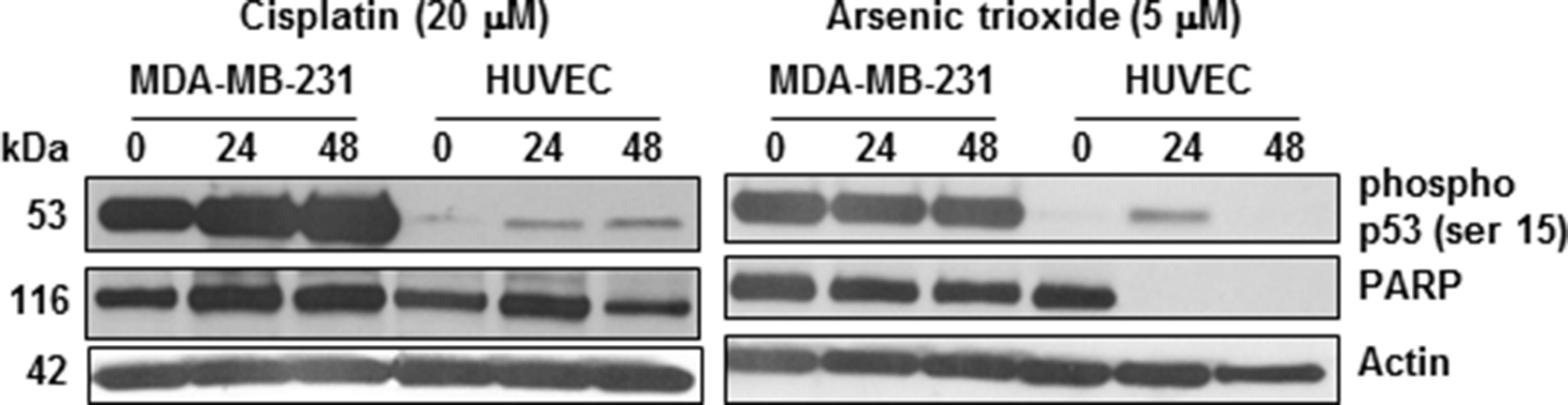
Sensitivity of HUVECs but not tumor cells to cisplatin and ATO is associated with phosphorylation of p53 MDA-MB-231 tumor cells and HUVECs were treated with 20 μM and 10 μM cisplatin and ATO respectively. Treatment with both drugs caused decrease in PARP expression and serine 15 phosphorylation of p53 in HUVECs but relatively no change in both in the MDA- MB- 231 cells.

FDA approved, Metal-based drugs such as ATO and cisplatin administered at high concentrations for effective tumor cell killing often present dose-limiting side effects. However, their encapsulation in nanoliposomes bound to receptors for specific cell targeting allows for their prudent use in improvement of anti-cancer efficacy. Molecular profiling with antibody arrays and the ingenuity pathway analysis in our earlier studies indicate the enhanced activation/phosphorylation of proteins associated with the apoptotic or stress signaling in response to radiation enhanced targeting of arsenic and cisplatin loaded liposomes in 3D murine TTA and their orthotopic implants to occur via phosphorylation of the functional p53 in endothelial cells [25]. In this study have designed an experiment to demonstrate a similar effect in the sensitivity of two different human cell types to arsenic and cisplatin. MDA-MB-231, a typical human TNBC cell line, fails to achieve an IC50 at cisplatin concentration of 30 μM [18]. Similar studies with ATO demonstrate a 12–15 μM IC50 for MDA-MB-231 cells [37, 38]. Figure 5 demonstrates a significant decrease in the expression of intact poly ADP-ribose polymerase (PARP) (116 kda), indicative of more cell death, in endothelial cells (HUVEC) but not the MDA-MB-231 TNBC cells incubated at much lower concentrations of cisplatin (20 μM) and ATO (5 μM). Literature suggests that the constitutively phosphorylated mutant p53 observed in several cancer cell types, escapes the Murine Double Minute 2 protein (MDM2) and stabilizes in the tumor cells [39, 40], enabling them to evade cell death. However, the transient activation of wild type p53 in response to pharmacological stimulus or stress triggers a flexible cascade of gene expression that determines whether a cell type will undergo cell cycle arrest or programmed cell death [41–43]. Proteolytic cleavage of PARP by caspases is a universal phenomenon observed during programmed cell death induced by a variety of apoptotic stimuli including those that trigger activation of p53 [44–46]. It has thus been used in this study as a readout for evaluation of apoptotic cell death. Our results demonstrate that while the p53 defective MDA-MB-231 cells despite displaying profuse constitutive phosphorylation at the serine 15 residue, show no decrease in intact PARP, the functional p53 in HUVEC undergo phosphorylation by 24 hr with an associated decrease or complete absence of full length PARP in response to either drug treatments, indicating that the sensitivity of different cell types to the two drugs is associated with the on activation/phosphorylation of p53. Based on this differential sensitivity of cell types contributing to the tumor formation, the nanotherapeutic strategy augmented by radiation exposure is so designed to target cytotoxic payloads of ATO and cisplatin that while increasing the dual drug concentration at the tumor site, will also create a microenvironment pernicious for survival of tumor cells. Efforts are now underway to delineate the p53-dependent and independent cell death/ damage via the intercellular crosstalk in response galectin-1 targeting in the tumor and its microenvironment utilizing our 3D TNBC tumor model [25].

### Radiation-enhanced therapeutic targeting of galectin-1 in the murine TNBC model developed from orthotopic implant of 3D TTA in nude mice

**Figure 6:**
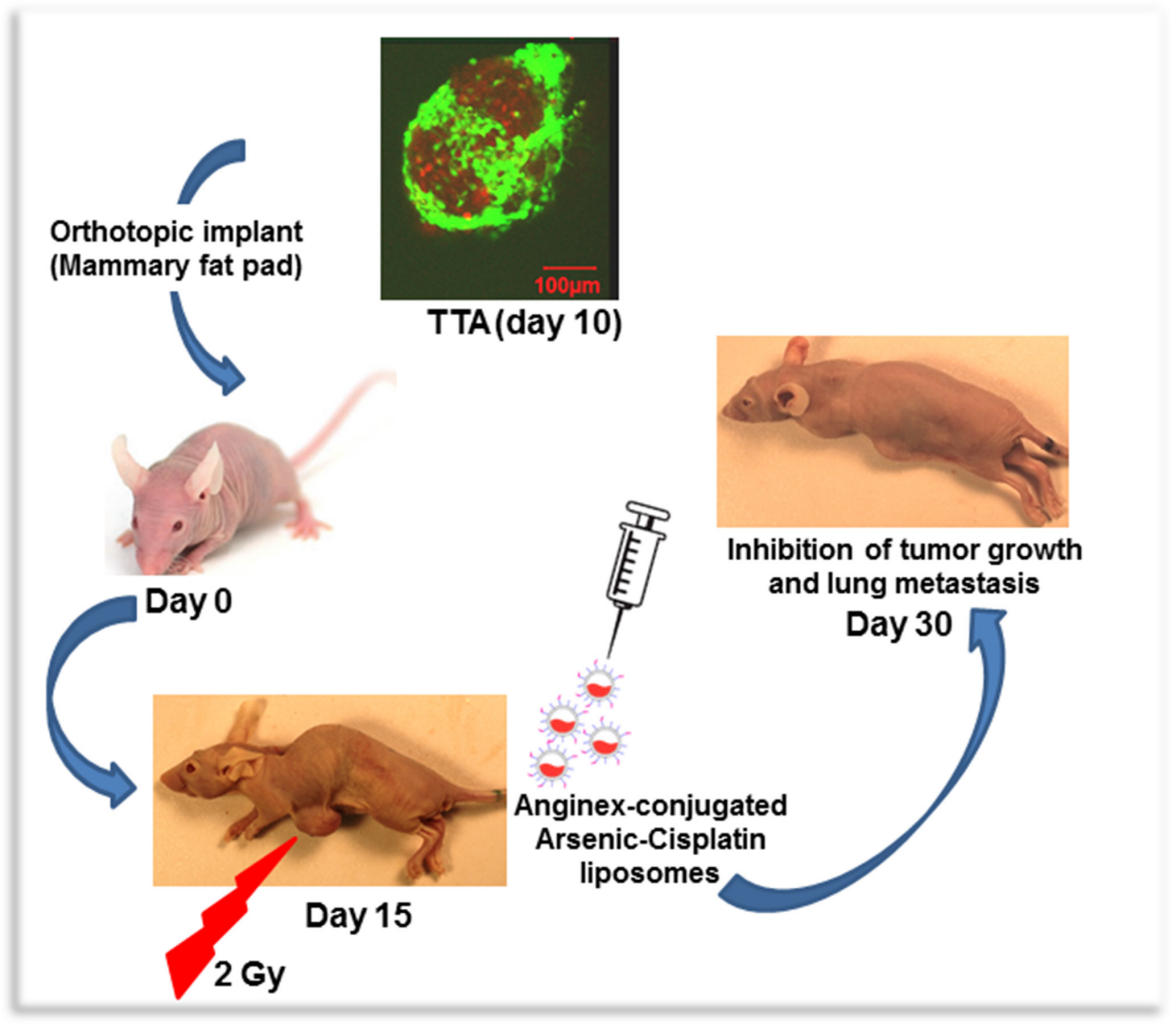
A Schematic representation of radiation enhanced nanotherapeutic targeting in the orthotopic tumor originating from Tumor tissue analogs (TTA) recreating TNBC and its microenvironment

The complex organization of tumor microenvironment has been shown to be a critical component of response to therapeutic interventions in cancer [31, 47–50]. The field of cancer therapy is therefore in the midst of a major paradigm shift from an approach primarily focused on tumor cell killing to strategies that also targets the tumor microenvironment [51]. Preclinical tumor models that more appropriately represent the tumor and the malignant stroma such as the 3D TNBC model that we have developed from TTA implants in athymic nude mice enhance the ability to study devise target-based therapeutic interventions. Our recent studies demonstrate targeting of irradiated tumor endothelial cells via radiation-induced stromal enrichment of Galectin-1 using Anginex conjugated liposomes encapsulating ATO and cisplatin. The platinum-arsenic loaded liposomes are very stable and are designed to release the drug only when internalized and processed through receptor-mediated endocytosis. Figure 6 illustrates the radiation enhanced tumor-targeting of anginex conjugated dual drug-(ATO and cisplatin) loaded liposomes using the murine tumor model that better represents the microenvironment and structural characteristics of TNBC.

### The combinatorial nanotherapeutic strategy inhibits tumor growth and metastasis in the murine tumor model for TNBC

**Figure 7:**
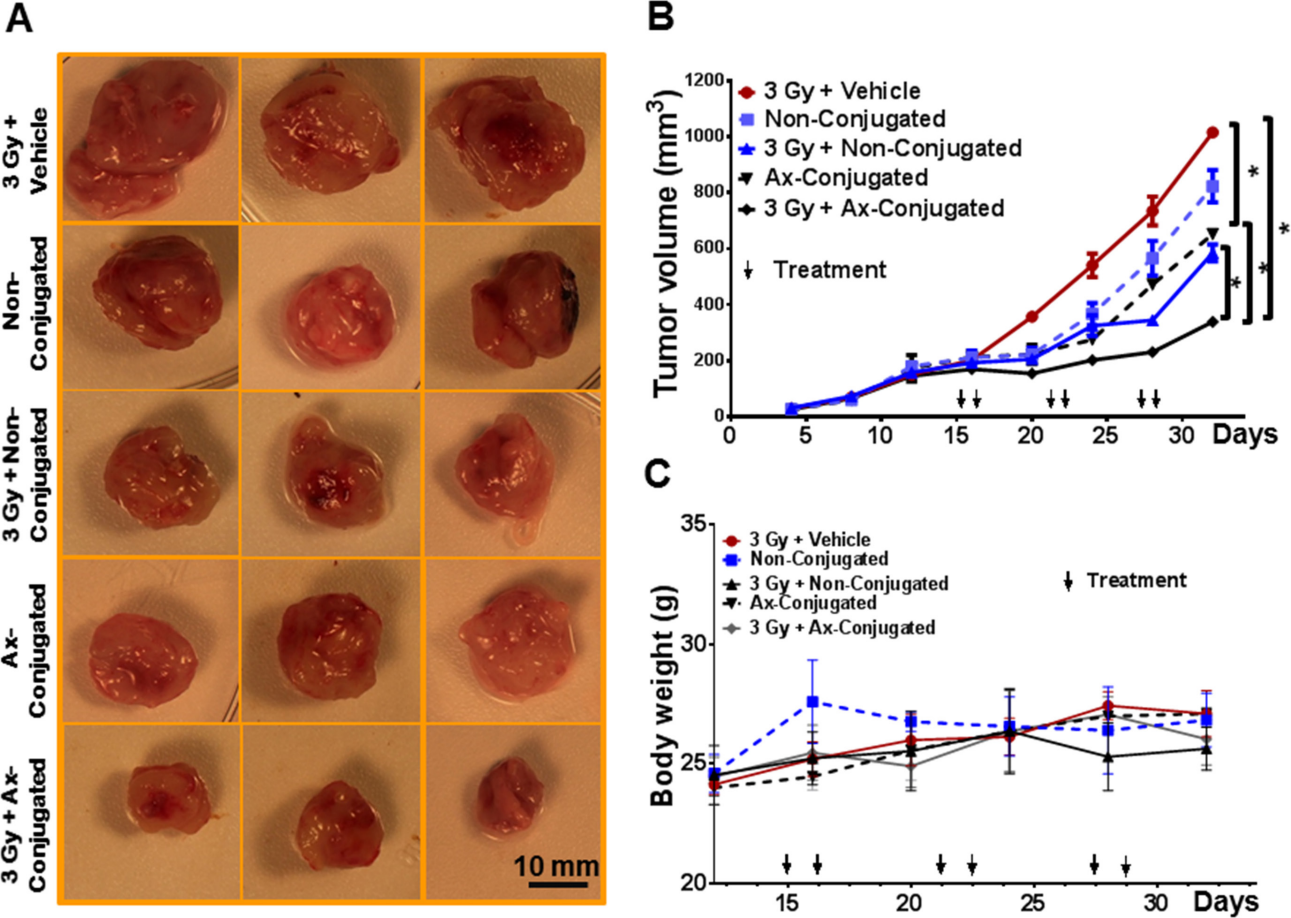
The combinatorial nanotherapeutic strategy inhibits tumor growth with no significant change in body weight in the murine TNBC model (**A**) Representative images of mammary tumors post treatment in female athymic nude mice with orthotopic implants of TTA. 14 days after surgical implantation of TTA in the mammary fat pad, mice were randomized into five treatment groups: Radiation + empty liposomes (3 Gy + vehicle), non-conjugated (ATO/cisplatin loaded) liposomes (Non-conjugated), Radiation +non-conjugated liposomes (3 Gy + Non-Conjugated), anginex-conjugated (ATO/cisplatin loaded) targeted liposomes (Ax-conjugated), Radiation + anginex-conjugated targeted liposomes (3 Gy + Ax-Conjugated). Agents were delivered 2 hrs post-radiation twice at an interval of 24 hr via tail vein injection and the regimen was repeated three times with a gap of 4 days as indicated. (**B**) Graphical representation of tumor growth in mice with orthotopic xenografts of TTA. (**C**) No significant weight loss was observed in any treatment group compared with radiation + vehicle-treated mice. Results indicated as mean ± SD (*N* = 3 mice/group). *p < 0.005 (vehicle vs non-conjugated and vehicle vs Ax-conjugated, non-conjugated vs ax-conjugated nanoparticle treatment with and without radiation).

The therapeutic targeting of anginex-conjugated (4 mg ATO/kg, 2.5 mg cisplatin /kg), non-conjugated (4 mg ATO/kg, 2.5 mg cisplatin /kg) was evaluated in non-irradiated and irradiated tumors (~200 mm^3^) originating from TTA implants in the mammary fat pad of athymic nude female mice. Empty liposomes administered to mice bearing irradiated tumors served as control. Our initial observations of TTA implanted orthotopically in the mammary fat pad of mice resulted in large tumors (~1500 mm^3^) with aggressive metastasis to the lungs. The rapid progression of the disease required the mice to be sacrificed within 20–24 days [25]. Expecting a similar outcome in tumor bearing mice when treated with vehicle alone we opted to leave this treatment group from the experiment designed to understand the response to a treatment regimen for ~3 weeks. Further, initiating the nanotherapeutic treatment regimen when the orthotopic tumors are ~200 mm^3^ (was also based on the rationale and our previous studies to ensure the formation of the tumor-associated stroma for targeting in the murine TNBC model [25]. While non-conjugated liposomes and anginex conjugated liposomes inhibited growth of irradiated and non-irradiated mammary tumors respectively by ~40%, tumor growth was significantly reduced by ~80% in irradiated tumors treated with anginex-conjugated liposomes. The tumor growth in the non-irradiated mice treated with non-conjugated liposomes was reduced only ~20% (Figure 7A–7B). Combined treatment of radiation and targeted nanoparticles was well tolerated by the mice with no significant difference in body weight when compared to radiation control (Figure 7C).

### The combinatorial nanotherapuetic strategy inhibits metastasis to the lung and results in increased accumulation of arsenic and cisplatin in the tumor tissue

**Figure 8:**
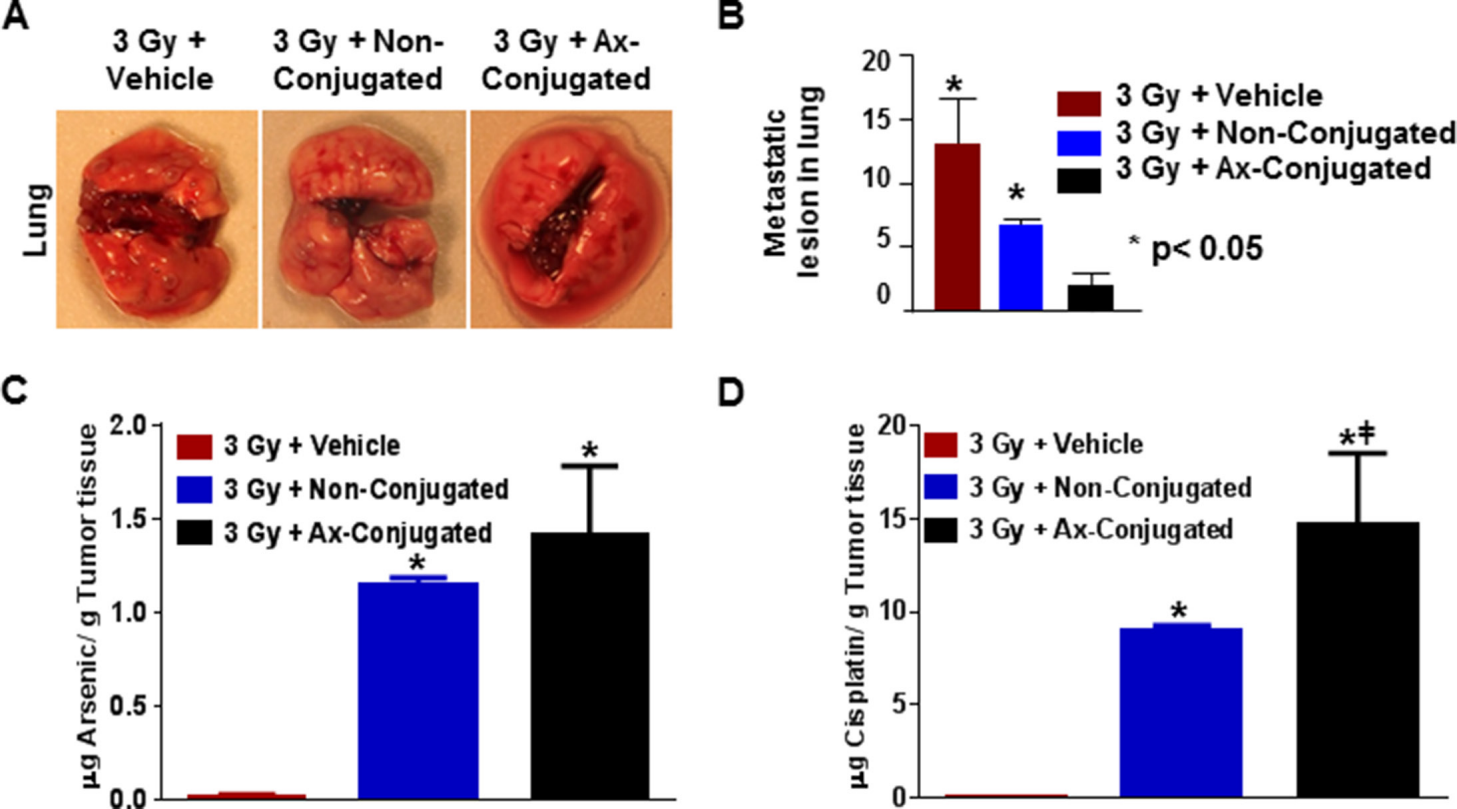
The combinatorial nanotherapeutic strategy results in inhibition of lung metastasis with increase in Arsenic and cisplatin accumulation in the murine tumor model for TNBC (**A**) Representative images metastatic lesions in diseased lungs post treatment in female athymic nude mice with orthotopic implants of TTA randomized in three treatment groups: Radiation + empty liposomes (3 Gy + vehicle), Radiation +non-conjugated (ATO/cisplatin loaded) liposomes (3 Gy + Non-Conjugated), Radiation + anginex-conjugated (ATO/cisplatin loaded) targeted liposomes (3 Gy + Ax-Conjugated). Agents were delivered 2 hrs post-radiation twice at an interval of 24 hr via tail vein injection and the regimen was repeated three times with a gap of 4 days as indicated. (**B**) Graphic representation of lung metastasis in mice bearing tumors originating from TTA implants. Results indicated as mean ± SD (*N* = 3 mice/group).*p < 0.05 (vehicle vs non-conjugated and Ax-conjugated nanoparticle treatment). Athymic nude mice with orthotopic implants of TTA after completion of the 6 treatments in the treatment regimen were sacrificed and tumor tissues were analyzed for arsenic (**C**) and cisplatin (**D**) by ICP-MS. Results indicated as mean ± SD (*N* = 3 mice/group) with *p < 0.001 for for arsenic cisplatin acquisition at the end of the study in mice with irradiated tumors treated with vehicle vs non-conjugated and vehicle vs Ax-conjugated nanoparticle. The #*p* < 0.05 was also significant for cisplatin but not arsenic uptake in irradiated mice treated with non-conjugated vs ax-conjugated nanoparticles.

Consistent with enhanced tumor efficacy, our study on invasion of the disease to distant organs revealed that metastasis to the lung was much reduced in the irradiated animals treated with the targeted liposomes (Figure 8A–8B). In our previous study we have demonstrated an increased uptake of the targeted nanoparticle with higher accumulation of arsenic and cisplatin 24 hr post treatment by the irradiated tumor tissue in the orthotopic implants of the TTA in nude mice [25]. These results established the radiation-enhanced targeting of stromal galectin-1 by the anginex-conjugated nanoparticles in the murine tumor model for TNBC. In this study, using the same TNBC model we have quantified the arsenic and cisplatin accumulation in the tumor tissue at the completion of the treatment regimen by ICP-MS. While the uptake of both arsenic and cisplatin in the irradiated tumors of mice at the end of the treatment regimen with the anginex-conjugated nanoparticles is higher than with non-conjugated nanoparticles, it is not as significant (Figure 8C–8D). It is now a universally accepted fact that all nanoscale particulate carriers including targeted and passively targeted liposomes are distributed to the target cells via the same passive distribution mechanism, by means of the enhanced permeability and retention effect [52]. An increased uptake of targeted liposomes by the diseased tissue, occurs as a consequence of the increased receptor-mediated uptake of liposomes, containing the entrapped drug, by the target cell [53]. The significant increase in arsenic and cisplatin accumulation in the irradiated tumor tissue after a single dose of the anginex-conjugated nanoparticle is possibly owing to the relatively faster clearance of the non-targeted nanoparticles from the intratumoral space [25]. However, repeated dosing as in this study may have increased the accumulation of the non-conjugated nanoparticles without affecting the drug uptake by the target cell and the subsequent improvement in therapeutic response. We also observe higher accumulation of cisplatin in the tumor tissue (9 μg/g tissue or higher) at the completion of the treatment regimen of radiation in conjunction with targeted or non-targeted nanoparticles (Figure 8C). Cisplatin undergoes aquation to form [Pt(NH_3_)_2_Cl(OH_2_)]^+^ and [Pt(NH_3_)_2_(OH_2_)_2_]^2+^ once inside the cell, intercalating between the DNA strands forming the cisplatin-DNA adducts [54]. The half-life of free ATO on the other hand falls between 10–48 hr (Agency for Toxic Substance & Disease Registry, 2007) and therefore we observed a notably lower concentration of arsenic in tumor tissues (1–2 μg/g tissue) post-treatment (Figure 8D).

The tissue residence of arsenic and cisplatin at the end of the study in different organs of the treated mice by ICP-MS showed a similar trend as observed in the tumor tissues of higher accumulation of cisplatin in comparison to arsenic (Supplementary Figure S3). The kidney and liver were sites with maximum residence of arsenic and cisplatin. ICP-MS analysis of the brain tissue revealed minimum accumulation of the two drugs. Restricted entry of most biomolecules by the blood brain barrier [55] and the glomerular kidney or sinusoidal liver capillaries forming the fenestrated vascular bed are the possible reasons for the respective low and high acquisition of arsenic and cisplatin in these organs [56]. Systemic toxicity during the treatments, was assessed by H&E staining of tissue sections from different organs of the experimental mice (Supplementary Figure S4). The H&E images indicated that all major organs after treatments maintained their typical structural phenotypes and did not exhibit appreciable microscopic lesions, further confirming the minimal side effects and acceptable biocompatibility.

## DISCUSSION

Compelling evidence gathered over recent decades indicate galectin-1, to be amongst the unique repertoire of proteins overexpressed across a spectrum of human cancers. These include breast [57], colon [58], lung [59], head & neck [60], ovarian [61] and prostrate [62] carcinomas in addition to gliomas [63], Kaposi's sarcoma [64], myeloproliferative neoplasia [65] and Hodgkin lymphoma [66]. The overexpressed galectin-1 is localized both in the stroma surrounding the tumor cells and in the cancer associated endothelial cells [67–69]. Here we report an elevated expression of galectin-1 in clinical samples from TNBC patients and data collected from the Human protein atlas on ductal carcinoma in women between the ages of 27–40 years (Figures 1 and 3). The galectin-1 expression profile in tumor and benign tissue sections of ductal carcinomas was also comparable to VEGF, a multifunctional cytokine, most clinically exploited for vascular targeting in tumors (Figure 1). To develop a tumor specific galectin-1 targeting delivery system that enhances the effectiveness of the nanotherapeutics, it was essential for us to assess the differential expression of galectin-1 in normal and tumor tissue. In accordance with the results from studies on other cancer associated malignancies, we also found, galectin-1 to be consistently overexpressed in the tumor-associated stroma of TNBC patients with significantly lower expression in benign breast and normal tissues from other organs (Figures 1–3). The recruitment of endothelial cells to form new blood vessel is an important step in the development, invasion and metastatic colonization of proliferative neoplasms such as the TNBC [70]. The targeting of transformed tumor cells has several limitations owing to their inherent drug resistance and highly heterogeneous malignant cell population [47–49]. While most chemotherapy is capable of killing tumor cells efficiently, it lacks the ability for selective targeting [71, 72]. Conversely, many anti-angiogenic agents, while not able to control tumor growth, possess the ability to selectively target the location and process of tumor blood vessel formation [73, 74]. The current study capitalizes on the radiation enhanced targeting of the antiangiogenic, anginex peptide to the galectin-1 enriched tumor microvasculature [24, 75] and the existing technique of liposomal encapsulation of chemotherapy to design a novel therapeutic approach for selective and effective inhibition of tumor growth and metastasis in TNBC. Ongoing efforts in the laboratory are directed to perform in-depth investigations for understanding the mechanistic underpinnings of radiation-augmented galectin-1 surge in the 3D TTA that enable enhanced binding of anginex-conjugated targeted liposomes.

While there are no specialized guidelines for TNBC treatment, single or multi-agent chemotherapy continues to remain the mainstay in the treatment of TNBC patients. Owing to concerns for significant toxicity, radiation when used is delivered sequentially following systemic chemotherapy. The benefits of radiation therapy in locoregional control and recurrence in breast cancer with improved survival [76] has sparked a recent interest to analyze the role of radiation in breast conserved therapy and locoregional recurrence in TNBC [77–79]. Interestingly, our studies have revealed a further induction of the stromal galectin-1 in response to radiation exposure in 2D, 3D and various preclinical tumor models [24, 25] and (Figure 4), indicating the possibility of combining radiation with targeted nanochemotherapy and reduced side effects. Furthermore, though ATO and cisplatin are identified as a synergistic drug combination in lung and squamous cell carcinomas [80, 81], the use of metal-based cytotoxic agents in standard clinical treatments of tumors is currently limited because (i) they cause acute systemic toxicity and cannot be administered at effective concentrations, (ii) are unstable in circulation and (iii) are subject to multiple mechanisms of drug resistance. The co-encapsulation of these drugs in nanocarriers overcome most of these challenges. When bound to receptors for specific cell targeting these nanovesicles are at a further advantage, as they are taken up by receptor-mediated endocytosis, thereby avoiding drug efflux by P-glycoprotein and overcoming drug resistance [82].

Evidence supports a major subgroup of TNBC originating from epithelial cells is genomically unstable with mutated p53 [83]. Our study using a p53 defective human epithelial TNBC cell line, MDA-MB-231 and a p53 functional human endothelial cell type, HUVEC, indicates the sensitivity to ATO and cisplatin in different human cell types to be regulated by activation/phosphorylation of p53 phosphorylation (Figure 5) and is similar to the effect observed in our *in vitro*/*in vivo* 3D murine TNBC models [25]. This led us to design a two pronged active targeting strategy augmented by radiation exposure (Figure 6): that while increasing the dual drug concentration at the tumor site, creates a microenvironment unconducive for tumor cell survival. ATO encapsulated in nanovesicles was expected to facilitate effective tumor-targeted delivery by causing vascular damage [84, 85] and overcoming resistance of tumor cells to conventional cisplatin chemotherapy. The controlled release of cisplatin from the tumor endothelium targeted liposomes was expected to further enhance the therapeutic efficacy of the drug delivery system by simulating the effect of low-dose metronomic chemotherapy [86]. In this study we investigate the therapeutic outcome of radiation enhanced tumor-targeting of nanosized ATO and cisplatin chemotherapy to the galectin-1 enriched malignant stroma. Data obtained from this study suggests the means to exploit clinically relevant radiation dose for concurrent receptor mediated enhanced delivery of chemotherapy while limiting overall toxicity (Figure 7). The minimal weight-loss and lack of observable signs of toxicity in organ histology associated with liposomal treatment in conjunction with radiation is attributed to the stability of the drug payload in physiological and acidic conditions, and so despite typical liposomal accumulation in the liver and other tissues there is minimal toxicity (Figure 7D). Since treatment options available for TNBC are limited, the ability of such a novel nanotherapeutic system to deliver combined therapy while limiting toxicity has the potential to improve survival outcome in TNBC patients and accelerate drug development.

## MATERIALS AND METHODS

### Cell lines and culture

4T1-mCherry is a red fluorescent protein-expressing murine metastatic mammary carcinoma cell line that closely mimics the triple negative subtype of human breast cancer [87]; was a kind gift from Dr. D. D. Schlaepfer (University of California, San Diego, CA). 2H11, murine tumor endothelial cell line [88], and C166-GFP, a murine green fluorescent protein-expressing endothelial cell line, were purchased from ATCC (Manassas, VA). Murine embryonic fibroblasts (MEF) were a kind gift from Dr. V. Rangnekar (University of Kentucky, Lexington, KY). The cell lines were routinely cultured in high glucose DMEM containing 10% (v/v) fetal bovine serum and 100 IU/mL penicillin, 100 IU/mL streptomycin at 37°C, 5% CO_2_, and 95%.

### Chemicals and reagents

Arsenic [Arsenic (III) oxide] and Cisplatin [cis-Diamineplatinum (II) dichloride] were purchased from Sigma-Aldrich, Inc. (St. Louis, MO). Anginex peptide was purchased from AAPPTEC (Louisville, KY). The antibodies, galectin-1 (Sigma #HPA000646) and (R&D AF-1152) were used for immunohistochemistry of clinical and murine tumor tissue samples respectively. The antibodies, p53 phospho (ser^15^) (#9284) and poly (ADP-ribose) polymerase [PARP] (#9532) for western immunoblotting were purchased from Cell Signaling Technology. Lipids used for liposome preparation were purchased from Avanti Polar Lipids (Alabaster, Al).

### Tumor tissue analogs (TTA) for orthotopic implant in nude mice

4T1-mcherry tumor cells, C166-GFP endothelial cells and murine embryonic fibroblasts (MEF) were used to generated single or multicell type 3D cultures in “hanging drops” of media (Dulbecco modified Eagle medium with 10% fetal bovine serum and antibiotic mix) as previously described [50]. Briefly, single a cell suspension of 4T1-mcherry cells, C166-GFP cells and MEF cells in equal proportion (3000 cells/20 μL) was dispensed on the inside of the lid of each well of a 48-well cell culture plate (Greiner Cellstar, BioExpress, Kaysville, UT). The growth of TTA was monitored over time until day 14 in a hanging drop of medium, following which they were subject to radiation exposure and treatment with liposomes. The 3D co-cultures/TTA were subsequently transferred to optically clear Greiner repellent plates for imaging and analysis of the treatment response over a period of 8–10 days.

### Animals

All animal procedures were approved by the University of Kentucky Animal Institutional Care and Use Committee (IACUC). Female, athymic nude mice (Crl:NU(Ncr)-*Foxn1nu*) obtained from Charles River (6–12 weeks old, 20–22 gm) were purchased from Harlan's Laboratories (Haslett, MI). All experimental mice were housed in sterile environmental conditions of the University of Kentucky's Division of Laboratory Animal Resources (DLAR) and provided sterile food and water *ad libitum*.

### Orthotopic breast cancer model

Mice were anesthetized with isoflurane (1–4% vaporizer) or Ketamine/Xylazine, IP, 100/10 mg/kg respectively. Two TTA generated from 3D cultures of tumor cells, endothelial cells and fibroblasts as described in [25] were subcutaneously implanted by a small incision (2–4 mm) bilaterally into the fat pad of the 3rd mammary gland and sutured with tissue adhesive (3M VetBond, St. Paul, MN) Tumor growth was recorded by caliper measurement after a recovery period of 1–2 days. Mice were euthanized at study endpoint by isoflurane overdose. Tumor tissues and lungs were excised for further analysis.

### Immunohistochemistry for galectin-1 expression

### In clinical samples of tumor and benign tissue fromTNBC patients

Six cases of triple negative breast cancer from formalin fixed surgical archives of the University of Kentucky were selected and 4-micron thick sections were cut from each and dried overnight at 58°C. Slides were deparaffinized and hydrated stepwise followed by heat-induced epitope retrieval (HIER) in high pH antigen retrieval solution (Dako) at 110°C for 20 minutes using a Biocare Medical Decloaking Chamber. Slides were incubated in Galectin-1 antibody (Sigma #HPA000646) diluted at 1:400 for 30 minutes at room temperature followed by incubation with polymer bound anti-rabbit secondary for 30 minutes (Dako Envision+). Staining was visualized by incubation with 3, 3′-diaminobenzidine (DAB) chromogen (Dako, CA) for 3 minutes and slides were lightly counterstained with hematoxylin.

### In orthotopic tumors originating from TTA in athymic nude mice

Formalin fixed paraffin embedded tissue was sectioned at 4-microns and dried overnight at 58°C. Slides were deparaffinized and hydrated stepwise followed by heat-induced epitope retrieval (HIER) in high pH antigen retrieval solution (Dako) at 110°C for 20 minutes using a Biocare Medical Decloaking Chamber. Slides were incubated in Galectin-1 antibody (R&D AF-1152) diluted at 1:50 for one hour at room temperature followed by incubation with polymer bound anti-goat secondary for 30 minutes (Vector Laboratories Immpress kit). Staining was visualized by incubation with 3, 3′-diaminobenzidine (DAB) chromogen (Dako, CA) for 5 minutes and slides were lightly counterstained with hematoxylin.

### Evaluation and quantification of immunohistochemistry

All slides were digitalized on an APERIO^®^ ScanScope (Leica Biosystems) and were evaluated on an APERIO^®^ ImageScope (Leica Biosystems)using the positive pixel counting algorithm, which scores the stains as negative, weak-positive, medium, and strong. This algorithm also measures the percentage of positivity by area and the average intensity of positive staining. HScore for each case of benign and tumor tissue sample from TNBC patient, based on the percentage and intensity of staining was determined. HScore takes into consideration the intensity of the staining and the percentage of positive cells per the formula: HScore = 1 × (% light staining) + 2 × (% moderate staining) + 3 × (% strong staining). HScores range from 0 to 300 [89].

### Hematoxylin and eosin staining and imaging

Hematoxylin and eosin (H&E) staining was performed by staining the cryostat sections of TES with Harris hematoxylin (aluminum potassium sulfate, hematoxylin, absolute alcohol, mercuric oxide, and glacial acetic acid) followed by 1% acid alcohol and, subsequently, 1% eosin. Images were taken at 40X magnification using a Nikon Ti E upright microscope with a Cool SNAP HQ2 CCD camera (Tokyo, Japan) and processed with NIS-Elements basic research software.

### Tumor/Cell X−Ray irradiation

Radiation exposure was given using the Varian TrueBeam System (Varian medical systems, Palo Alto, CA) X-Ray machine set at a radiation dose rate 1.018 ± 0.10 Gy/ min at 150 kV and 6.6 mA. Typical radiation exposure in all experiments was at 3 Gy. Mice were anesthetized with Ketamine/Xylazine, IP, 100/10 mg/ kg, and radiation was administered with a custom cut lead shielding covering the animal, except for the tumor-bearing region.

### Whole cell lysates and immunoblot analysis

Whole cell extracts were prepared by suspending cells in 0.25 ml of lysis buffer (25 mM HEPES, pH 7.5, 0.5% 498 J Mol Med (2013) 91:497–506 sodium deoxycholate, 5 mM EDTA, 5 mM dithiothreitol, 20 mM glycerophosphate, 1 mM Na3VO4, 50 mM NaF, 1% Triton X-100, 20 μg/ml aprotinin, 50 μg/ ml leupeptin, 10 μM pepstatin, 1 μM okadaic acid, and 1 mM phenylmethylsulfonyl fluoride). The lysates were incubated at 4°C with gentle agitation on a rocker plate (Mini Mixer, Benchmark Research Products, NY) for 1 h and cell debris was removed by centrifugation (15 min at 12,000 × g). The protein concentration in the supernatant was determined usingthe bicinchoninic acid (BCA) protein assay kit (Pierce). Immunoblotting was performed using 30 μg of protein/lane and standard polyvinylidene fluoride membrane transfer followed by probing with the respective antibody and chemiluminescent detection of bands.

### Synthesis and characterization of Anginex-conjugated liposomes

The untargeted liposomes were synthesized using the aquo-cisplatin concentration gradient as previously described [90]. In these studies, untargeted liposomes were comprised of disteroylphospatidyl choline, cholesterol, and distearoylphosphatidyl ethanolamine-mPEG_2000_ at a molar ratio of 0.5/0.46/0.04, respectively, with additional 0.1 mol% DiD (1,1′-Dioctadecyl-3,3,3′,3′-Tetramethylindodicarbocyanine Perchlorate) fluorescent dye (Life Technology). The lipids were produced at large scale using techniques described previously [91, 92] Anginex-azide was synthesized by modifying the protein at the N-terminus using azide-dPEG_4_-NHS (Quanta Biodesign). Alkyne functional lipids were synthesized and inserted into pre-formed liposomes as previously described [92]. Anginex-azide was reacted with the alkyne-liposomes at 25-fold molar excess compared to concentration of liposomes. Unconjugated anginex was removed by tangential flow filtration. Phospholipid, arsenic and platinum concentration was determined by ICP-OES and particle size was measured by dynamic light scattering (Malvern Zetasizer). Nanob were characterized by ICP-OES, using a matrix of 3% nitric acid, 2% acetic acid and water. Details are provided in the methods described earlier [25]. Briefly, the liposomes were diluted into matrix, then analysed by ICP-OES. Size was determined by dynamic light scattering in the Keck Biophysics Core Facility, using a 514 nm laser equipped with a Brookhaven BI-200 goniometer and BI-9000 high speed correlator. The modification of the liposome formulation with anginex to target galectin-1 overexpressed on irradiated endothelial cell surface occurs in three convergent steps. First, the anginex is modified with a heterobifunctional crosslinker containing an azide group. Next, drug-loaded liposomes are modified with an alkyne-containing lipid molecule, creating alkyne-modified reactive liposomes. The alkyne liposomes and the azide Anginex are then cross-linked using a copper-catalyzed alkyne-azide cycloaddition “click” reaction [25].

The resulting liposomes are stable at 4°C for 6 months without aggregating, precipitating or loosing therapeutic potency. Drug release from the liposomes is triggered by thiol-containing compounds, such as glutathione.

### Tail vein injection

The animals were anesthetized as described above. DID [Dioctadecyl-tetramethylindodicarbocyanine] labeled liposomes (4 mg ATO/kg, 2.5 mg cisplatin/kg) were administered at a volume of 150–200 μl with a 27 G needle in the lateral tail vein of mice bearing the subcutaneous mammary fat pad tumors ~200 mm^3^ in size.

### Inductively coupled plasma-mass spectrometry

The quantitative analysis of Platinum and Arsenic in tumor tissue samples of tumors and other organs was accomplished via ICP-MS of acid digested samples using a Thermo XSeries II ICP-MS (Thermo-Fisher Scientific, Waltham, MA) as described [91]. Briefly, Platinum and Arsenic quantification in tumor tissues was accomplished via ICP-MS of acid digested samples using a Thermo XSeries II ICP-MS (Thermo-Fisher Scientific, Waltham, MA). Specifically, samples were prepared by adding 200 μL of nitric acid (HNO_3_, 70%, Aristar Plus Grade, BDH) and 50 μL of hydrochloric acid (30%, Aristar Plus Grade, BDH) to pre-weighed tissues and incubated at 80°C for 8 hrs for complete sample digestion. Multi-element internal standard (Inorganic Ventures, Christiansburg, VA) and filtered deionized H_2_O (18.2 MΩ·cm) were added, producing a final ICP-MS sample of 4% (v/v) HNO_3_, 1% HCl, and 5 ng/mL multi-element internal standard.

### Statistical analysis

Data are summarized as mean ± SD for each experimental group. Two-sample *t*-test or analysis of variance was employed for two-group and multiple group comparisons, respectively, with contrast generated from the ANOVA model to perform pairwise comparisons. Paired *t*-test was employed for comparison of Galectin-1 H-score for paired normal and tumor samples. Finally, linear mixed model was employed for comparison of tumor volume over time. Statistical analysis was performed using SAS version 9.4.

## SUPPLEMENTARY MATERIALS FIGURES


