# Supplementary material for: Radiation-enhanced therapeutic targeting of galectin-1 enriched malignant stroma in triple negative breast cancer
Source: Oncotarget. 2016 May 19;7(27):41559–74. doi: 10.18632/oncotarget.9490 (PMC5173078; doi:10.18632/oncotarget.9490)
Supplement: Supplementary file 1 [file oncotarget-07-41559-s001.pdf]

Radiation-enhanced therapeutic targeting of galectin-1 enriched malignant stroma in triple negative breast cancer

Supplementary Materials

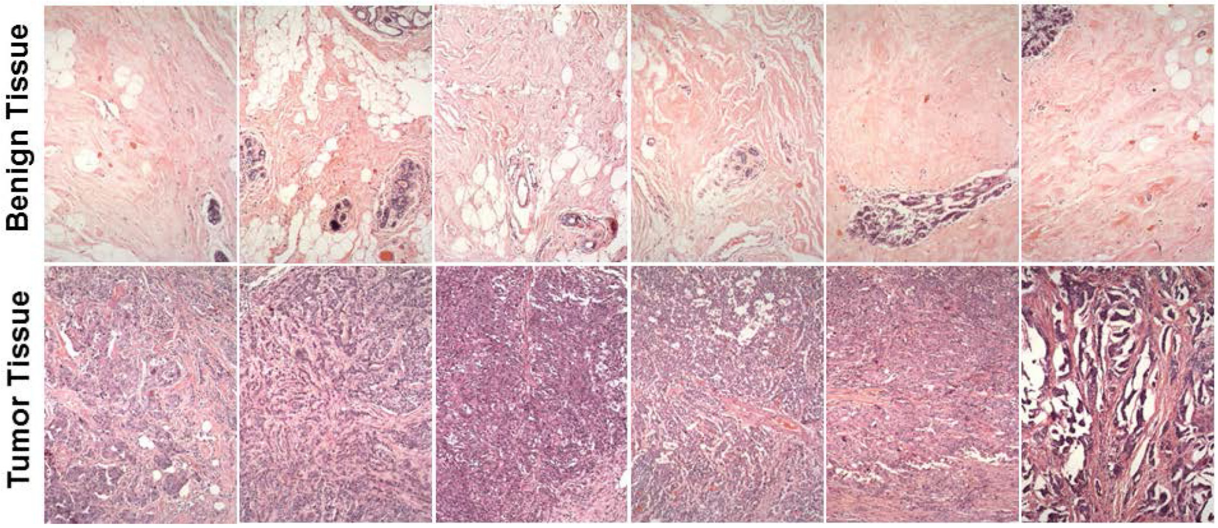

Supplementary Figure S1: Histology of tumor and benign tissue sections from clinical samples of TNBC patients. Tumor and benign tissue sections that were immunostained for galectin-1 (Figure 3) were also stained with H & E to determine the histology of the respective tissues. The images are at 100X magnification.

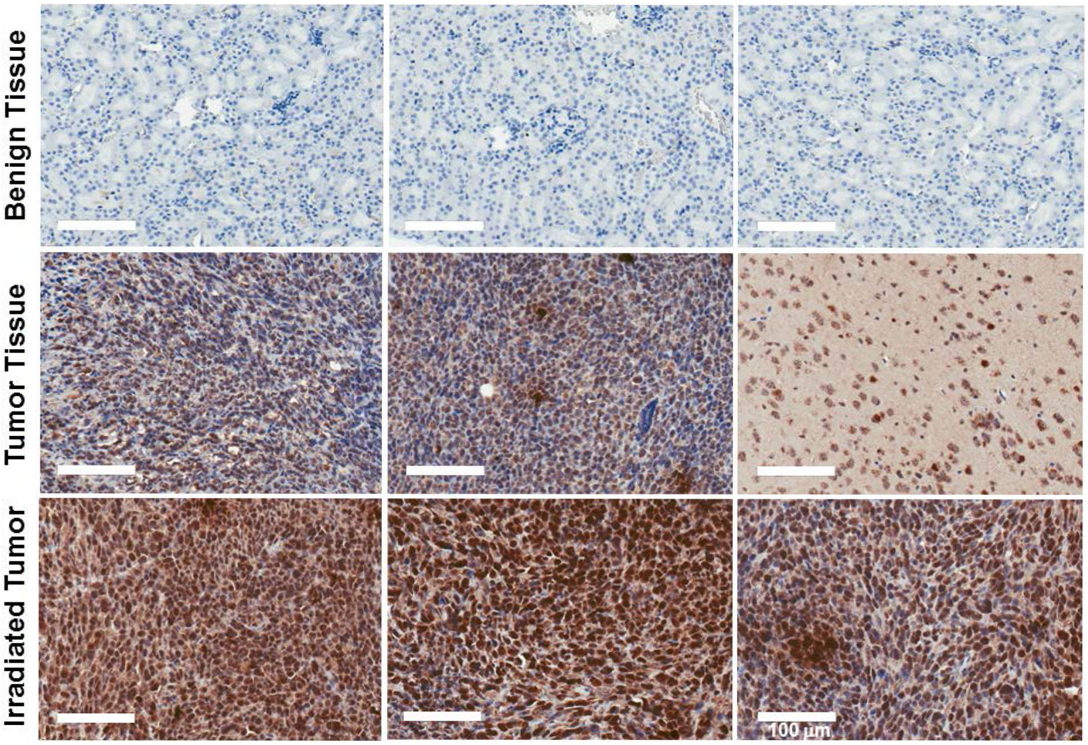

Supplementary Figure S2: Radiation augmented galectin-1 expression in orthotopic tumors originating from TTA. Iimmunohistochemistry for galectin-1 in benign breast (upper panel, BT), tumor (middle panel, TT) and irradiated tumor (lower panel, IT) tissue originating from orthotopic implants of TTA in mice 72 hours post-radiation exposure of 2 Gy.

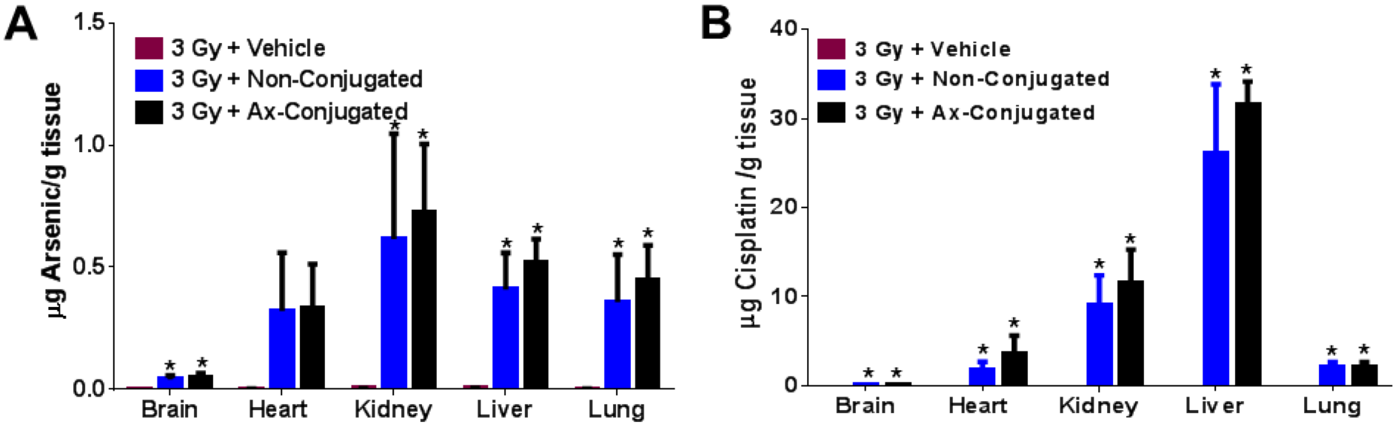

Supplementary Figure S3: Tissue residence of arsenic and cisplatin and biocompatibility of the treatment process. The tissues from organs such as the Brain, Heart, Kidney and Liver and Lung of tumor bearing mice after completion of the treatment regimen were analyzed for accumulation of arsenic (**A**) and cisplatin (**B**) by ICP-MS. Results indicated as mean ± SD. ($N = 3$ mice/group). *$p < 0.05$ compared to vehicle. Non-conjugated vs ax-conjugated (Non-significant).

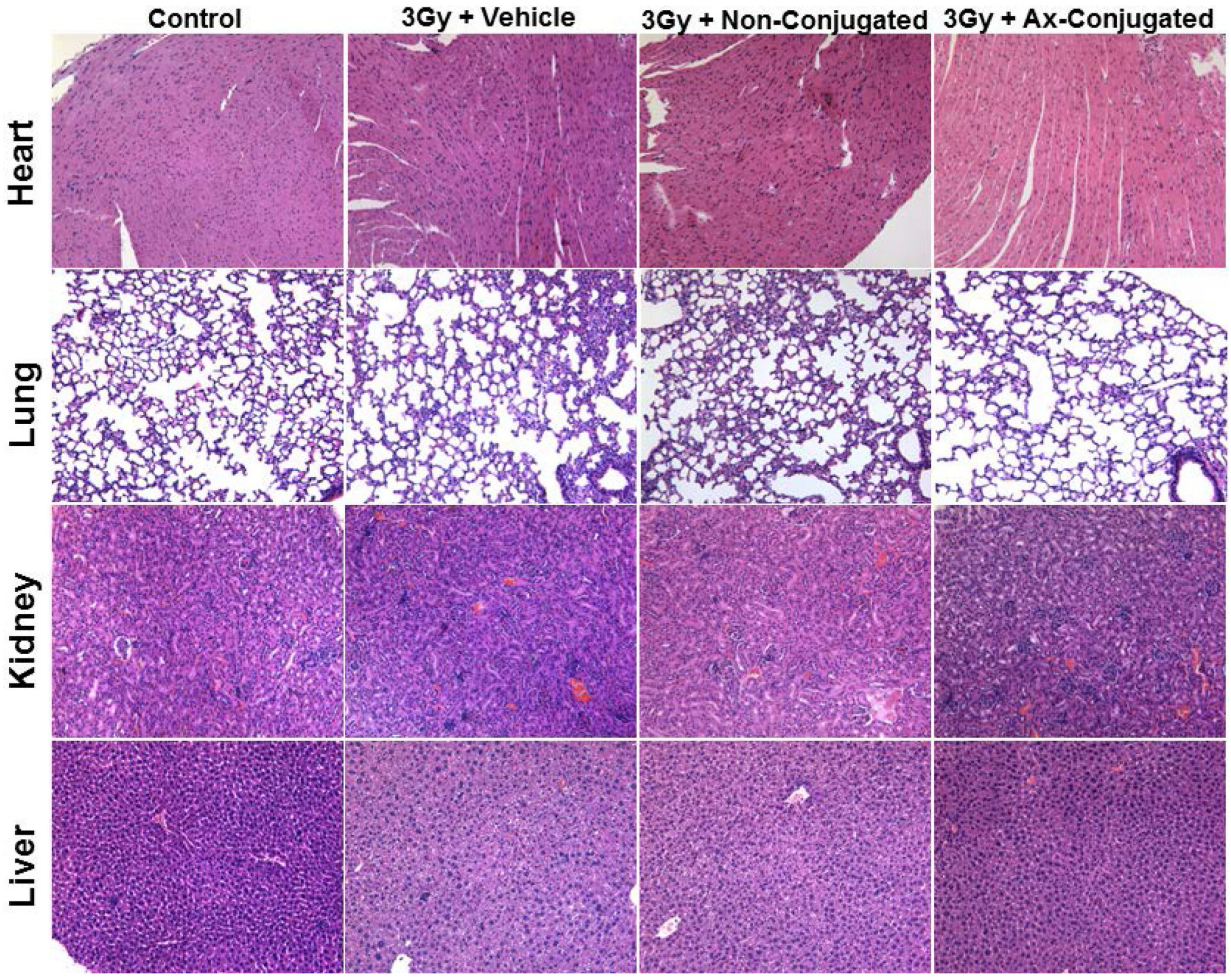

Supplementary Figure S4: Biocompatibility of the treatment process. The organ histology was assessed by staining the tissue sections for H & E. Shown here are the representative fields of H & E stained Heart, Lung Kidney and Liver tissue. ($N = 3$ mice/group).
